# Thyroid surgery – Does the scar matter?

**DOI:** 10.20945/2359-3997000000381

**Published:** 2021-06-29

**Affiliations:** 1 Memorial Sloan Kettering Cancer Center New York Department of Surgery New York USA Head and Neck Service, Department of Surgery, Memorial Sloan Kettering Cancer Center New York, New York

Boog and cols. from São Paulo, Brazil, have reported the impact of scar on thyroidectomy patients (1). They have reviewed the aesthetic impact of scars on the lives of the patients. This is a retrospective analysis of 98 patients determining the scar impact through a qualitative questionnaire and categorizing three levels of dissatisfaction. Of 98 patients, 96 (98%) reported experiencing no functional or visual discomfort with their scars. There were 2 women who were unsatisfied to a moderate category. It would have been interesting if the authors had interviewed these two patients as to their reasons behind dissatisfaction and the examiner’s impression about their scars. They have also discussed their impressions about conventional thyroidectomy against other extra cervical or scarless thyroidectomy. Their conclusions are consistent with the current literature regarding thyroidectomy scars. There may be some issues related to the methodology used by the authors, but the conclusions remain that most of the patients are quite happy with the thyroidectomy scars. I would like to take this opportunity to congratulate the authors for reconfirming our beliefs and would like to add some pertinent pointers about thyroidectomy scar.

There is always debate about the size of the incision; however, the wounds heal in a transverse fashion and there is hardly any cosmetic impact between smaller and larger incisions. What is critical is to get adequate exposure during surgery based on the size of the tumor, location of the tumor, the patient’s configuration, and age of the patient. In a younger patient we prefer higher incision, as the scar gets pulled down over a period of time. The incision should be marked preoperatively based on the location of the cricoid cartilage and cervical creases. Surgery through small incision may be challenging, especially stretching the skin edges, and may require freshening the edges.

As the old aphorism goes, “The bold surgeons make small incisions, the scared surgeons make big incisions, but the good surgeons make adequate incisions”. The incision is best made around or just below the cricoid cartilage and generally not recommended above the cricoid. If the patient requires a neck dissection, the incisions should be extended into the neck like a necklace at the level of cricoid. There is no need to make an apron or J-shaped incision. The surgical incision heals very well and is cosmetically readily accepted by the patients and their loved ones. The operating surgeon should spend more time in meticulous closure of the wound with appropriate subcuticular stitches. The platysma should be approximated well, which reinforces the skin and avoids wound separation. We generally use 5-0 monocryl subcuticular. Some surgeons use subcuticular prolean to be pulled out 3 days after surgery.

One needs to be extremely careful in location and closure of the incision in African American patients who have higher probability of developing a keloid or hyperplastic scar. The incision in the appropriate skin crease literally hides the incision in most of the patients. There are very few patients who are unhappy or who complain about the scars and most of the scars continue to get better in the postoperative period up to 9 to 12 months. Certain ancillary measures are quite helpful such as skin lotions-vitamin E, silicon, Scarguard, etc. Several patients demand wound closure by plastic surgeon even though it is not necessary, as essentially they perform the same surgical technique as thyroid surgeons.

Felix and cols. reported a high level of scar satisfaction (92%) after conventional thyroidectomy (2). Obviously some patients may be self-conscious of their incision. Kurumety and cols. published patient-reported data on thyroidectomy scar perception (3). They concluded older age and more than 2 years after surgery as predictive factors for scar perception. They also concluded “the impact of postoperative thyroidectomy neck appearance on quality of life to be mild and transient and returns to preoperative levels after 2 years”.

There are newer surgical techniques of scarless thyroidectomy such as transaxiliary robotic thyroidectomy or the most recent addition of transoral thyroidectomy. These newer techniques are clearly surgical nuances with impact of technology; however, whether these are absolutely essential to avoid a scar will depend on variety of factors such as surgeon’s experience, learning curve, patient’s acceptance, and the cost. Prior to these newer techniques almost every thyroidectomy patient accepted the scar as is and we may need to revisit the whole issue of scar versus no scar. The newer techniques may have some application in highly selected patients. We need to revisit the extracervical scarless procedures in view of excellent patient satisfaction with standard open thyroidectomy.

Overall satisfaction rate in thyroidectomy scar is quite high and most of the patients learn to live with their scar and quite often enjoy it very much.
